# Stress Management Apps With Regard to Emotion-Focused Coping and Behavior Change Techniques: A Content Analysis

**DOI:** 10.2196/mhealth.6471

**Published:** 2017-02-23

**Authors:** Corinna Anna Christmann, Alexandra Hoffmann, Gabriele Bleser

**Affiliations:** ^1^ Junior research group wearHEALTH Department of Computer Science University of Kaiserslautern Kaiserslautern Germany

**Keywords:** mHealth, mobile health, relaxation

## Abstract

**Background:**

Chronic stress has been shown to be associated with disease. This link is not only direct but also indirect through harmful health behavior such as smoking or changing eating habits. The recent mHealth trend offers a new and promising approach to support the adoption and maintenance of appropriate stress management techniques. However, only few studies have dealt with the inclusion of evidence-based content within stress management apps for mobile phones.

**Objective:**

The aim of this study was to evaluate stress management apps on the basis of a new taxonomy of effective emotion-focused stress management techniques and an established taxonomy of behavior change techniques.

**Methods:**

Two trained and independent raters evaluated 62 free apps found in Google Play with regard to 26 behavior change and 15 emotion-focused stress management techniques in October 2015.

**Results:**

The apps included an average of 4.3 behavior change techniques (SD 4.2) and 2.8 emotion-focused stress management techniques (SD 2.6). The behavior change technique score and stress management technique score were highly correlated (r=.82, *P*=.01).

**Conclusions:**

The broad variation of different stress management strategies found in this sample of apps goes in line with those found in conventional stress management interventions and self-help literature. Moreover, this study provided a first step toward more detailed and standardized taxonomies, which can be used to investigate evidence-based content in stress management interventions and enable greater comparability between different intervention types.

## Introduction

Chronic stress has been shown to influence people’s physical and mental well-being [1,2]. For example, evidence is growing that stress is related to depression, cardiovascular disease, human immunodeficiency virus or acquired immunodeficiency syndrome, upper respiratory tract infections, asthma, herpes viral infections, autoimmune diseases, wound healing, and tumor progression [2,3]. Additionally, chronic stress and health are linked indirectly through stress-related behaviors such as smoking, sedentary lifestyle, poor eating habits, alcohol and drug abuse, as well as insufficient therapy adherence [2,4].

Although the effects of stress depend on the timing, duration, and to some extent on the interaction between genes as well as the previous exposure to environmental adversity [5], an individual’s well-being depends not only on his or her exposure to stress, but also on the way he or she copes with this stress. Two broad types of stress management can be distinguished: problem-focused and emotion-focused coping [6]. Problem-focused stress management refers to methods attempting to alter the relationship with the environment, whereas emotion-focused stress management methods aim at reducing, tolerating, or eliminating stress sensations.

Stress management group interventions usually use a multitechnique approach. The variability between studies and technique descriptions does not, however, allow conclusions about which combination of techniques should be used [7]. The same applies to self-help books in this context (see [8] for a bibliography and reading recommendations). Most of them present a broad range of coping techniques. Although it is unclear how many coping techniques should be adopted to achieve a maximum improvement of stress-related symptoms, it has been shown that not all coping techniques work equally well for every individual (eg, the effects of hypnosis are highly dependent of a person’s suggestibility [9]). Moreover, certain stress management techniques are especially useful for reducing specific kinds of symptoms [10].

Besides self-help literature, psycho-technology mobile apps have emerged as a useful complementary tool in psychotherapy [11]. The recent mHealth trend offers a new and promising approach to support the adoption and maintenance of appropriate health behavior. As mobile phone users can be reached anytime and anyplace [12], apps can be used as a platform for behavioral interventions [13]. Furthermore, mHealth apps allow the usage of gamification aspects that can potentially increase users’ motivation [14]. Following this idea, mobile phone–based stress management interventions could result in savings for the health care system [15], provided that they are effective.

However, little is known about the usage of specific coping strategies in current stress management apps. Although there are first indications that at least some of them might be effective, for example, StressEraser [16-20] or AEON [21], most stress management apps have not been evaluated yet [22,23]. So far, 3 reviews have been published. Lee et al [22] come to the conclusion that current stress management devices show controversial theoretical underpinnings and a lack of systematic evaluation. Plaza et al [23] investigated app objectives within meditation apps and reported that only 56-61% of these apps were in fact devoted to meditation. Coulon et al [24] conducted the first analysis of stress management apps with regard to 7 evidence-based stress management strategies, transparency in app development, and functionality of the app interface in spring 2015. Mindfulness and meditation as well as diaphragmatic breathing and seeking social support were used most frequently in these apps. Visualization and imagery, active coping, problem solving, and cognitive restructuring were less common. Only half of the samples included evidence-based content, as well as acceptable usability and functionality. This study provided a first impression regarding the use of evidence-based content in current stress management apps including a brief list of problem-focused and emotion-focused coping strategies.

However, to the best of our knowledge, there is no established taxonomy with regard to emotion-focused stress management strategies. Therefore, a corresponding taxonomy including clear definitions for each strategy was developed for this app analysis. In addition to the emotion-focused stress management strategies that have been investigated by Coulon et al [24] (namely breathing exercises [25-27], progressive muscle relaxation [26,28], meditation or mindfulness [29-31], and visualization or guided imagery [26,32]), the following evidence-based strategies were identified during a thorough literature review: autogenic training [33], biofeedback [26], emotional freedom technique or acupressure [26,34], euthymic methods [35], hypnosis or self-hypnosis [36], (self-)massage [37], and physical stress relief techniques such as yoga [38] or tai chi [39]. As stress sensations can also be influenced by some types of music [40,41], sounds of nature [42], nutrition [43], and sport [44], these aspects were also included (see Table 1 for an overview and definitions). By considering a broader range of established methods, this approach interestingly allows for a more extensive investigation on the usage of emotion-focused stress management strategies.

Based on the taxonomy developed by Abraham and Michie [45], additional evidence-based behavior change techniques have been investigated in this review. This taxonomy has already been used in previous health app analyses [46-50], revealing that the usage frequency of evidence-based behavior change techniques varied with mean scores on a low to moderate level. Furthermore, it has been shown that stress management (which is included as a behavior change technique in this taxonomy) is used only rarely [47,51], thereby underpinning that stress management only seems to play a subordinate role in current health apps. More importantly, using the same taxonomy helps to compare the results of this app review with those of other health apps.

Although problem-focused coping is not the main focus of this taxonomy [45], it is interesting to note that some problem-focused coping strategies for stress management are nevertheless addressed, namely planning of social support and social change [52], time management [53], self-monitoring [54], and goal setting [55].

This was the first study to investigate the usage of evidence-based content in current stress management apps based on such detailed taxonomies. Not only this approach reveals problems in current stress management apps, the detailed and standardized taxonomy of emotion-focused stress management strategies can also be used in further stress management research to increase comparability between different intervention types.

## Methods

### Selecting Apps for Review

**Figure 1:**
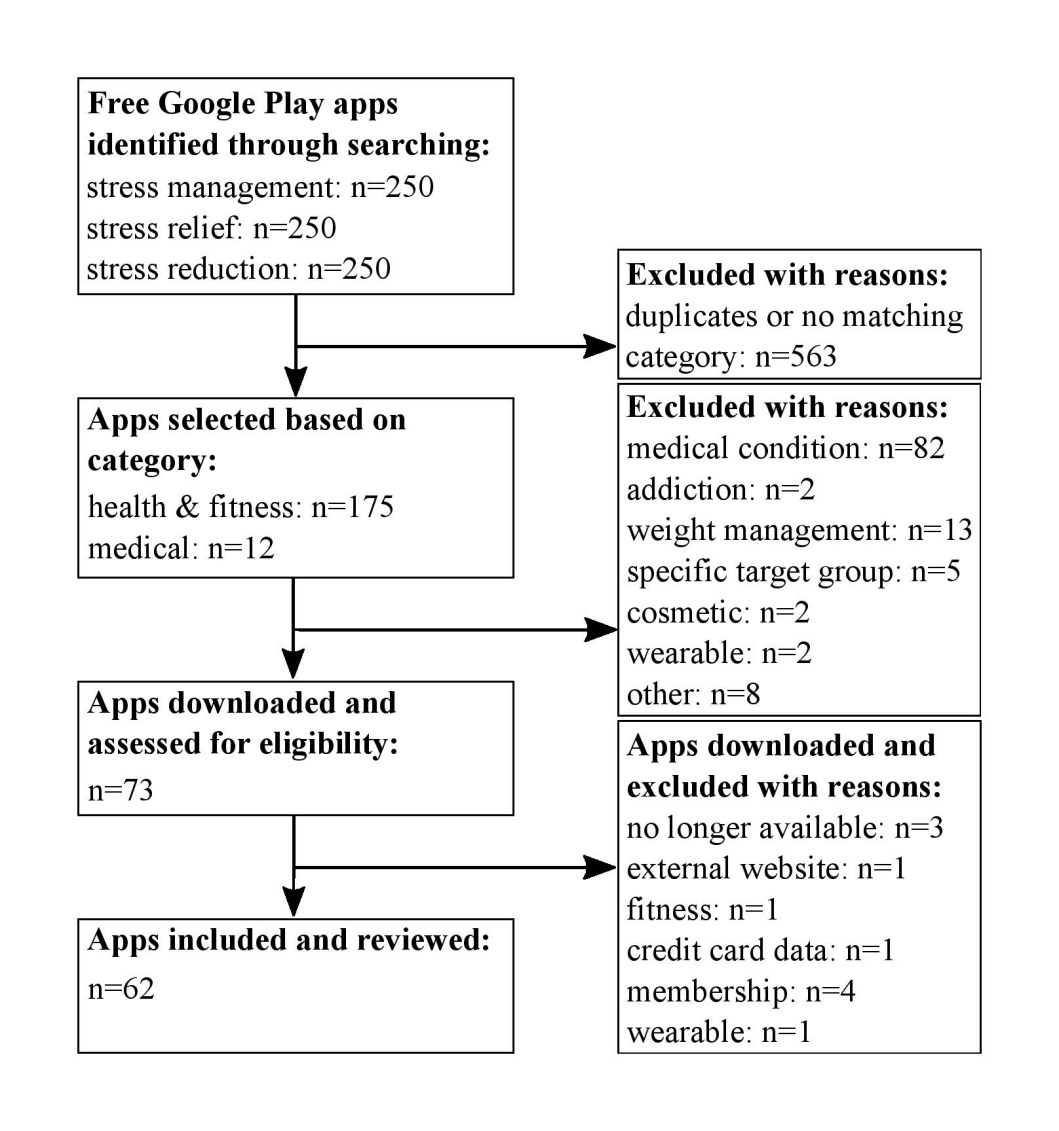
Flowchart for schematic overview of the selection process for stress management apps. The resulting sample comprised 62 apps.

As Android has become the most frequently used mobile phone operating system on the global market [56] and systematic reviews for stress management apps from Google Play have not been published yet [24], this review only included apps, which were available through Google Play in October 2015. Apps were identified using the search terms “stress management,” “stress reduction,” and “stress relief”. For each search term, the first 250 free apps were checked regarding the following inclusion criteria (see Figure 1 for a schematic overview of the selection process): (1) Apps had to be provided in the “Health & Fitness” or “Medical” categories of Google Play to exclude apps that focus on “Entertainment” (eg, mini-game activities), “Beauty,” or “Music & Audio.” In line with this idea based on app descriptions, only apps were chosen that target stress management and well-being; (2) To ensure applicability for a broader range of people, the respective apps should target healthy adults and not specific groups, medical conditions, or weight management; specifically because apps targeting a specific group (eg, children, specific medical conditions) have different requirements compared with stress management apps for healthy adults; (3) Apps that require membership of a company were excluded for the same reason. Instead the focus was put on free apps, considering the fact that most apps in the categories “Health & Fitness” (90%) and “Medical” (86%) are provided for free in Google Play [57]; (4) Apps that require an additional wearable were also left out, as most wearables are still scarcely accessible to the general public [58]; (5) Finally, this review included only English apps to ensure a broader accessibility. Following this procedure, the only app that had also been investigated in the study of Coulon et al [24] was Breathe2Relax. At the time, about 50% of the apps used in this study were also available for iTunes.

### Procedure and Data Analysis

Apps that met all inclusion criteria were downloaded, installed, and tested using the Android Development emulator software of Android Studio version 1.3 (Google Inc) running Android OS 4.4 [59] by both raters in October 2015. At times, this approach was unsuccessful in regards to the presentation of some app content such as playback of audio or video files, download of data, and display of pages. Therefore, apps facing such difficulties were subsequently installed on a Nexus S mobile phone to examine the problematic features.

The 62 apps were downloaded and evaluated by two trained and independent raters (the second author and a graduate student of psychology) regarding two taxonomies: 1 for behavior change techniques and 1 for emotion-focused stress management strategies.

Each app allowed the users to progress at their own speed, allowing both raters to thoroughly check all features of the apps until it was apparent that no new features were going to be activated. The results of this review are based on content that was provided by the apps themselves. Information and features on websites linked within the apps were not considered.

### Evaluation Criteria and Instruments

The evaluation of behavior change techniques was based on an established theory-linked taxonomy. The full list of these techniques including detailed definitions can be found in the study by Abraham and Michie (2008) [45]. Stress management is included as one of those 26 behavior change techniques. Some aspects of problem-focused coping are also included in this taxonomy. However, it does not provide further insight into emotion-focused coping. Thus, a thorough literature review on evidence-based emotional stress reduction methods was conducted in major databases and revealed 15 emotion-focused stress management strategies and definitions (see Table 1).

**Table 1:** Effective emotion-focused relaxation techniques.

Technique	Definition
Acupressure or emotional freedom technique	Pressure is applied to specific points
Autogenic training	Six standard exercises: heaviness and warmth in the extremities, calm and regular function of the heart, self-regulation of respiration, soothing warmth in the upper abdomen (solar plexus) area, and agreeable cooling of the forehead
Biofeedback	Precise instruments measure physiological activity such as brainwaves, heart function, breathing, muscle activity, and skin temperature. These instruments rapidly and accurately “feedback” information to the user.
Breathing	Manipulation of breath movement or rate
Euthymic methods	Training of sensual behaviors that include positive experiences, such as the sense of smell, hearing, tasting, and feeling. These experiences take place in the real world, not in the imagination.
Food or nutrition	Healthy diet information (eg, which food to eat or which to avoid, how much to eat, drink,…)
Guided imagery or visualization	A facilitated exploration of an image of a safe, comfortable place that can or cannot be specific to the participant is involved including sensory recruitment (visual, auditory, olfactory, tactile, and kinesthetic)
Hypnosis or self-hypnosis	While being in a relaxed state, suggestions are voiced. The suggestion, no matter whether presented by oneself or another, is used to focus the conscious mind upon a single dominant idea.
Meditation or mindfulness	Focus of attention on body and surroundings or thoughts or food in the real world
Music	Strings of sounds, humming, or singing that form a melody
Muscle relaxation	The tensing and relaxing of muscle groups (eg, the legs, abdomen, chest, arms, and face) in a sequential pattern while focusing on the distinction between the feelings of the tension and relaxation
Physical stress relief techniques	Description of yoga, tai chi, stretching, qi gong,…. exercises
Self-massage	Massaging or rubbing of a specific body part
Sounds	Single and specific sounds (eg, nature sounds such as waterfalls, river flow, wind, bird song)
Sport	Description of how often and how long a specific sport (such as running, aerobics,...) needs to be performed

The inter-rater reliability was calculated according to Cohen’s kappa [60] as commonly used index for inter-rater agreement. To calculate the sum scores, disagreements of the two raters were treated as hits, resulting in a score between 0 and 26 for the behavior change techniques and between 0 and 15 for the stress management strategies.

## Results

Inter-rater agreement was acceptable for both, behavior change techniques (κ=.74) as well as emotion-focused stress management strategies (κ=.73). The sum scores for each app with regard to the behavior change techniques, the coping relevant behavior change techniques (stress management, prompt self-monitoring behavior, plan social support or social change, time management, and prompt specific goal setting), and emotion-focused stress management strategies can be found in the Multimedia Appendix 1.

An average of 4.3 behavior change techniques (SD 4.2, range 0-21 out of 26), 1.6 coping-related behavior change techniques (SD 1.29, range 0-5 out of 5), and 2.8 emotion-focused stress management strategies (SD 2.6, range 0-11 out of 15) was found. The highest sum score was found in “Mevii” by Thrive 4-7 with 21 behavior change techniques, 5 coping-specific behavior change techniques, and 9 emotion-focused stress management strategies. With regard to emotion-focused stress management, the highest sum score was found for “Stress Management Guide” by DHMobiApp with 11 different strategies. The behavior change techniques score and the emotion-focused stress management strategies score were highly correlated (r=.82, *P*=.01). There was also a correlation between the specific stress management strategies and the coping-relevant behavior change techniques (r=.69, *P*=.01).

**Figure 2:**
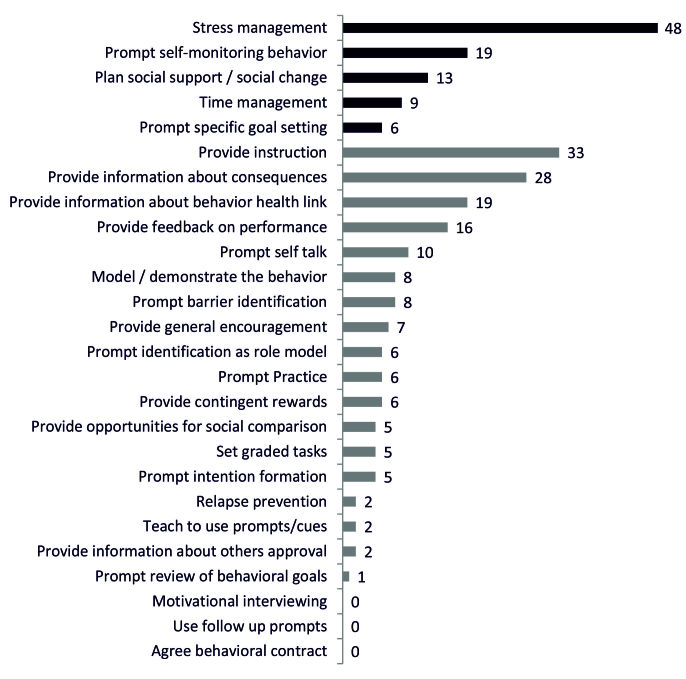
Behavior change techniques. Absolute frequencies of the 26 behavior change techniques used in the 62 apps, ranked by the most frequently applied techniques. Scoring followed the taxonomy of Abraham and Michie. Coping-relevant techniques are displayed in black and unspecific behavior change techniques are displayed in gray.

Figure 2 shows how often each behavior change technique was found in all apps [45]. Coping-relevant behavior change techniques are displayed in black, and the remaining techniques are displayed in gray. “Stress management,” “provide instruction,” and “provide information about consequences” were used most frequently, whereas “motivational interviewing,” “use follow-up prompts,” and “agree to behavioral contract” could not be found in any app.

**Figure 3:**
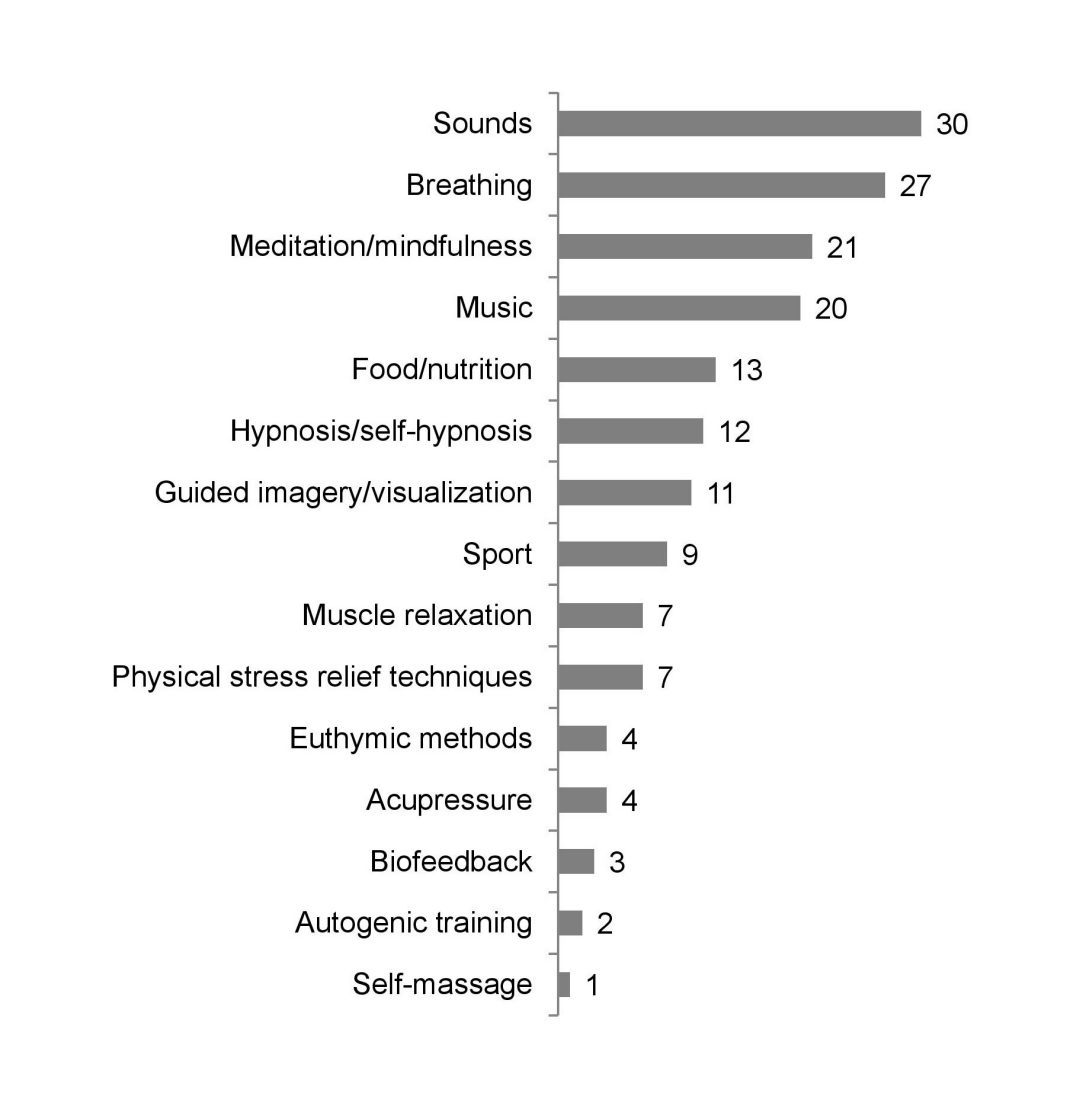
Stress management strategies. Absolute frequencies of the 15 emotion-focused stress management strategies used in the 62 apps, ranked by the most frequently applied techniques. Scoring followed the taxonomy described in Table 1.

Regarding the emotion-focused stress management techniques, “sounds,” “breathing,” “meditation or mindfulness,” and “music” were used most frequently. In contrast, recommendations regarding “food or nutrition,” “hypnosis or self-hypnosis,” “guided imagery or visualization,” “sport,” “muscle relaxation,” and “physical stress relief techniques” were used less frequently. Overall, “euthymic methods,” “acupressure,” “biofeedback,” “autogenic training,” and “(self-) massage” were hardly used (see Figure 3 for details).

The correlation analyses revealed that behavior change techniques and stress management strategies were frequently used in combination. These analyses and the absolute frequencies of each behavior change technique and stress management strategy did, however, not provide insights with regard to which methods were used simultaneously within one app. Therefore, association rules [61] were used to reveal clusters of jointly used methods within the apps. The rules are implications of the form: method X=> method Y, meaning that if method X is used within the app, method Y is used as well. Two indexes are assigned to each rule: support and confidence. Whereas support indicates how frequently the item set appears in the dataset, confidence indicates how often the rule has been found to be true.

The association rules revealed high co-occurrences for several behavior change techniques. For example, “provide instruction,” “plan social support or social change,” “provide information about consequences,” “provide information about behavior health link,” “prompt self-talk,” “prompt barrier identification,” “model or demonstrate behavior,” “prompt self-monitoring behavior,” and “provide general encouragement” were always used in combination with “stress management” (CI=100%). Moreover, “plan social support or social change,” “prompt self-talk,” and “prompt barrier identification” were always used in combination with “provide information about consequences” and “provide instruction” (CI=100%).

Concerning the specific stress management strategies, the analysis revealed that “muscle relaxation,” “autogenic training,” “biofeedback,” “guided imagery or visualization,” “meditation or mindfulness,” and “hypnosis or self-hypnosis” were frequently combined with “breathing” (CI 90-100%).

## Discussion

### Principal Findings

The aim of this study was to investigate the use of evidence-based content in free stress management apps in Google Play based on a new taxonomy of emotion-focused stress management strategies as well as an established taxonomy of behavior change techniques [45]. The analysis revealed an average of 2.8 emotion-focused stress management strategies with a high range from 0 to 11. This variability in the usage of different coping techniques goes in line with a review of group intervention studies on stress management in which the number of applied techniques also varies from 1 to more than 10 [7].

As the focus of this analysis was put on stress management apps, it is not surprising that stress management proved to be the most frequent behavior change technique in our sample. It should be mentioned, however, that 23% (14/62) of our sample did not include any emotion-focused stress management strategy at all. Some of these apps only provided information about stress without any further advice on how to cope with it. Others consisted only of playful elements or stress-related quotes. One app was a video with changing colors.

This result corresponds to a recent analysis of stress management apps from the Apple iOS App Store [24] in which no evidence-based strategy was found for one-third of the sample. In the study by Coulon et al [24], “mindfulness or meditation” (48%) and “diaphragmatic breathing” (17%) were found most frequently. In our sample, “mindfulness and meditation” were found slightly less frequently with 34%, whereas “breathing” exercises were found in nearly half of the sample (44%). Nevertheless, the criterion for breathing exercises was broader in this study, as it also included instructions that aimed at reducing the overall breathing rate. “Visualization and imagery” then again were found only in a small percentage of apps in both studies.

The comparability of results between this study and that of Coulon et al [24] shows that the usage of evidence-based content in apps from iTunes and Google Play apparently does not differ strongly between the two stores. Furthermore, it should be noted that about 50% of the apps that were used in this sample are also available on iTunes; this demonstrates that the choice of store only seems to play a subordinate role for this type of study. There was, however, hardly any overlap between our sample of apps and the one used by Coulon et al [24], as only one app (Breathe2Relax) was investigated in both samples.

Concerning behavior change, the apps in this analysis contained an average of 4.3 techniques. This mean behavior change techniques score was smaller compared with those found in other health app analyses using the same taxonomy but without special focus on stress management. These studies detected an average of 5 [51], 6 [50,62], or even 8 [47,63] behavior change techniques. This variation might be due to differences in app genres. Interestingly, in our sample, stress management and coping-focused behavior change techniques were used more frequently (on average 19 times per technique) than the remaining behavior change techniques (on average 8 times per technique). This indicates that although the absolute number of behavior change techniques was smaller compared with that of other health apps, the designers focused on techniques that were apparently relevant to stress management.

Self-regulation techniques, such as “self-monitoring,” “feedback,” and “goal setting,” have been reported as valued features within focus group discussions [64] and indeed are commonly used in weight management and fitness apps [47,51]. Although “self-monitoring” and “feedback” are considered as backbones of behavior change systems [63], they have only been discovered in a subsample of apps in this study: “Goal setting” was used only in 10% (6/62) of this sample.

From the association rules and the correlation analyses, it can be concluded that apps that use a broad range of emotion-focused stress management strategies also use a wider range of behavior change techniques. Moreover, the association rules revealed that most relaxation methods (90%-100%) in this sample of apps were combined with breathing exercises. This finding strengthens the content validity of the apps, as abdominal breathing exercises are the basic condition for mastery in other relaxation techniques [10].

There is, however, no clear consensus about how many and which behavior change techniques should be used in health behavior change systems [65]. Although 1 meta-analysis from 85 studies of Internet-based interventions based on more than 43,000 participants clearly speaks for an extensive use of different behavior change techniques—the number of techniques was related to greater effect sizes [66]—there was no indication of greater effect sizes with an increasing number of behavior change techniques in other studies [67,68].

The same applies to specific stress management techniques. Whereas there are recommendations about which techniques might be effective (see the Introduction for further details), there is no consensus about the absolute number and combinations that should be presented.

In general, most self-help books [8] contain a broad range of coping techniques. This might be explained by the fact that some of those techniques are especially useful for reducing specific kinds of symptoms [10]. As symptoms may change over time, it seems practical to provide people with a broad selection of coping strategies from which they can choose the most suitable ones according to their individual situation and specific symptoms. Nevertheless, one should note that although some of the apps in this sample allowed users to rate their symptoms and stress levels, none of those apps used that information to offer content that was specifically focused on the respective pattern of symptoms. Thus, this might be a promising approach for further health app designs.

Besides the obvious lack of evidence-based content in some stress management apps, it should be mentioned that the inadequate realization of behavior change techniques and stress management strategies is one of the largest threats in current stress management apps. One prominent feature is “provide information on behavior-health link.” Some stress management apps of this sample recommended the consumption of alcohol and medicine in order to reduce stress. One app instructed for frenzied and unsystematic breathing, which is related to stress rather than relaxation. These are only two examples of potentially harmful advice. Moreover, there are also first reports that some stress management strategies provided by stress management apps can evoke accompanying symptoms such as dizziness and drowsiness [17]. Besides, there can be disqualifying factors for stress management techniques: One example is autogenic training, which should only be applied under supervision of a physician in cases of diabetes, hypoglycemic conditions, or heart conditions [10]. These restrictions must be pointed out to the user prior to providing further instructions.

### Limitations

There are some limitations of this review that should be considered.

It is noteworthy that, although some aspects of problem-focused stress management such as time management, goal setting, and planning social support or social change are included in the behavior change technique taxonomy [45], the main focus of this analysis was put on emotion-focused stress management. Yet, future app analyses might extend the range of problem-focused strategies. The same applies to the taxonomy of behavior change techniques. However, although a more detailed and hierarchical version of the taxonomy is available [69], this study used an early version of the taxonomy [45] to increase comparability with prior health app analyses [46-50].

“Biofeedback” was only found in 3 apps. This might be due the fact that this technique often requires the additional use of a wearable device. In fact, only few measurements such as heart rate variability can simply be attained via the use of a mobile phone [70]. As most wearables are still scarcely accessible to the general public [58], apps that required additional hardware were not taken into consideration for this study. It might be for this reason that the usage of biofeedback has been underrepresented in this sample.

Finally, the analysis included only free apps. As prior health app analyses found that the use of evidence-based content was associated with the app prices [47,71] it cannot be ruled out that scores might be higher for paid apps.

### Conclusions

This study provides an extended overview of the usage of evidence-based content in mobile stress management apps. It depicts the first systematic review of current stress management apps available in the Google Play Store with regard to an established taxonomy of behavior change techniques [45] and a newly developed taxonomy of emotion-focused stress management strategies. This approach allowed a deeper insight into app content compared with prior app analyses of stress management [24] and mindfulness apps [23,72].

The broad variation of different stress management strategies that was discovered in this sample of apps corresponds to those found in conventional stress management interventions [7] and self-help literature [8]. As there is no consensus about how many and which combinations of techniques should be used, it is difficult to draw conclusions about overall app quality. The analysis, however, revealed a lack of use of evidence-based content in at least a subsample of apps.

This study provides a first step toward more detailed and standardized taxonomies to investigate evidence-based content in, for example, stress management interventions, self-help books, and stress-related mobile technology, enabling greater comparability between different intervention types.

## Appendix

Multimedia Appendix 1 App scores and relative ranking.
