# Supplementary material for: Stress Management Apps With Regard to Emotion-Focused Coping and Behavior Change Techniques: A Content Analysis
Source: JMIR Mhealth Uhealth. 2017 Feb 23;5(2):e22. doi: 10.2196/mhealth.6471 (PMC5344985; doi:10.2196/mhealth.6471)
Supplement: Multimedia Appendix 1 [file mhealth_v5i2e22_app1.pdf]

Multimedia Appendix. App scores and relative ranking

App Name	Category	BCTS[a]	BCT CS[b]	RTS[c]	BCTS rank	RTS Rank
Mevii by Thrive 4-7	health & fitness	21	5	9	1	2
Stress Management by Nasim	health & fitness	16	5	8	2	3
Health Foods Now by TopFreeAppsTips	health & fitness	13	4	7	3	4
Calm-IT Stress Relief by New Oceans	health & fitness	11	4	6	4	5
The Stress Surfer by Workforce Management Systems	health & fitness	11	4	6	4	5
3 Minute Stress Buster by Astute Software	health & fitness	10	3	4	5	7
Stress Relief Hypnosis by Shoonger	health & fitness	9	3	8	6	3
Get Out of Stress by AndroAppDevelopers	health & fitness	9	2	7	6	4
eSense Temperature by Mindfield Biosystems Ltd.	health & fitness	8	2	5	7	6
Pocket Clarity: Mindfulness by Three Pound	health & fitness	8	5	4	7	7
101 Ways to Be Healthy by Life Time Fitness	health & fitness	8	2	4	7	7
Stress Free Live Free by Davidandroidbro	health & fitness	8	3	4	7	7
Stress Management by EclipseBoy	health & fitness	8	1	4	7	7
Exercise - A Necessity by ACIW	health & fitness	8	2	2	7	9

Breathe2Relax by T2	health & fitness	7	4	4	8	7
Stress Relief Free Guide by AppBelle	health & fitness	7	3	3	8	8
Reiki-Energie by Dog Breeds Apps	health & fitness	6	3	6	9	5
Best Guided Meditation by Muhhas	health & fitness	6	1	6	9	5
StressLocator Free by Petr Kolman	health & fitness	6	1	2	9	9
O2CHAIR by INNOVZEN	health & fitness	6	3	2	9	9
Natural Stress Relief Hypnosis by Mastermind App	health & fitness	5	2	5	10	6
EFT Tapping Simulation by Mikhail Game Tech	health & fitness	5	2	4	10	7
INNOVZEN by INNOVZEN	health & fitness	5	1	2	10	9
RELAXATION OPTIMIZER by IntelaText	health & fitness	5	1	2	10	9
Preksha Meditation* by Preksha International	health & fitness	4	2	5	11	6
Stress Zapper by Green Infinity	health & fitness	4	1	2	11	9
Social BrainGym Lite by Brain Relax Technosoft	medical	4	1	2	11	9
Stress Management Guide by DHMobiApp	health & fitness	3	1	11	12	1
Free Meditation - Take	health & fitness	3	2	4	12	7

a Break by Meditation Oasis						
Motivational Quotes for Stress by Tiger Shark Pendekar	health & fitness	3	1	2	12	9
Stress Check by Azumio by Azumio Inc.	health & fitness	3	1	2	12	9
Reiki Heal by Devils Canon	health & fitness	3	2	0	12	11
Stress Reduction Audio by Mindware Consulting, Inc	health & fitness	2	1	6	13	5
Anti-stress Exercise by QUO?TE developers	health & fitness	2	1	3	13	8
Stress Flush by ENKI	health & fitness	2	1	2	13	9
Anti Stress by reism	health & fitness	2	1	2	13	9
Dr Yousef by Mobile App Company	health & fitness	2	1	1	13	10
Essential Oils for Stress by Almasi	health & fitness	2	2	0	13	11
Stressheads by YouthNet	health & fitness	2	1	0	13	11
Stress Check by kzk	health & fitness	2	1	0	13	11
Anti-Stress- by Aps Studios	health & fitness	1	1	2	14	9
Entspannende Musik by Aps Studios	health & fitness	1	1	2	14	9
Meditation Music by MeTapps	health & fitness	1	1	2	14	9
Regen ton by Aps Studios	health & fitness	1	1	1	14	10

Regen und Donner Geräusche by Aps Studios	health & fitness	1	1	1	14	10
Baoding Stress by GaMoon	health & fitness	1	1	1	14	10
Beste entspannende Musik by Aps Studios	health & fitness	1	1	1	14	10
Der Klang der regen by Aps Studios	health & fitness	1	1	1	14	10
Tiefe Meditation Musik by Aps Studios	health & fitness	1	0	1	14	10
Entspannungsmusik by Aps Studios	health & fitness	1	1	1	14	10
Wellen strand klingen by Aps Studios	health & fitness	1	0	1	14	10
WorkLIFE 365 by Lindsey Witmer Collins	health & fitness	1	1	1	14	10
Luftpolsterfolie - Stressabbau by ExaMobile S.A.	medical	1	1	0	14	11
5 Best Health Tips by eurekafox	health & fitness	1	1	0	14	11
POP POP by BIGTEXAPPS	health & fitness	1	1	0	14	11
AnandaYoga by ECHK Hong Kong	health & fitness	1	1	0	14	11
Best Self-Help Quotes by Waikiki Sky	health & fitness	0	0	0	15	11
Best Wisdom Quotes by Waikiki Sky	health & fitness	0	0	0	15	11
Dance Workout by Marinapps	health & fitness	0	0	0	15	11
Motivational Quotes	health & fitness	0	0	0	15	11

by Waikiki Sky						
stress relief reduce tensions by malcolm pugh	health & fitness	0	0	0	15	11
Sun Anywhere by Deux	health & fitness	0	0	0	15	11

[a]Total possible score = 26

[b]Total possible score = 5

[c]Total possible score = 15

BCTS, Behavior Change Techniques Score; BCTCS, Behavior Change Techniques Coping Score; RTS, Relaxation Techniques Score
